# The Paradox of Tik Tok Anti-Pro-Anorexia Videos: How Social Media Can Promote Non-Suicidal Self-Injury and Anorexia

**DOI:** 10.3390/ijerph18031041

**Published:** 2021-01-25

**Authors:** Giuseppe Logrieco, Maria Rosaria Marchili, Marco Roversi, Alberto Villani

**Affiliations:** 1 Residency School of Pediatrics, University of Rome Tor Vergata, 00165 Rome, Italy; giuseppe.logrieco@opbg.net; 2 Department of Emergency, Acceptance and General Pediatrics, Bambino Gesù Children’s Hospital, IRCCS, 00165 Rome, Italy; mrosaria.marchili@opbg.net (M.R.M.); alberto.villani@opbg.net (A.V.)

**Keywords:** adolescence, social media, anorexia, non suicidal self-injury

## Abstract

The literature shows that social pressure promotes non-suicidal self-injury (NSSI) Eating disorders, along with self-injury, are also favored by underregulated social media. Tik Tok is one of the most used social media platforms among adolescents. It has been shown that the time young children spend on this platform doubled during the lockdown. The theme of anorexia is very common on this platform. While most “pro-ana” (pro-anorexia) videos, where users exchanged advice on how to pathologically lose weight, have been censored by the application, other “anti-pro-ana” (anti-pro-anorexia) videos, officially aimed at raising awareness of the consequences of anorexia, have become increasingly popular. However, our case shows how even these safer videos paradoxically lead the users to emulate these “guilty” behaviors.

## 1. Introduction

Anorexia is the most prevalent eating disorder, primarily involving female adolescents, with a mortality rate 5 to 10 times higher than in the general population [1,2] and a high prevalence of non-suicidal self-injury (NSSI) [3]. A study also proved how the models of beauty displayed on social media can lead adolescents to develop an eating disorder [4]. The social restrictions due to Covid-19 have led adolescents to spend more and more time on social networks [5]. One of them is Tik Tok, a mobile application created in 2016 that in a few years reached more than 800 million users, at least 20% of which turned out to be teenagers [5,6,7]. This platform has been criticized on several occasions for poor content filtering and mediocre privacy protection [8]. The theme of eating disorders and self-harming behaviors is still present on the application, although disguised.

## 2. Case Report

We describe the case of a 14-year-old patient with a diagnosis of anorexia nervosa. Starting from April 2020 (a month after the Covid-19 related lockdown in Italy), the girl reported progressive restriction of food intake and increased physical activity (5 h per day of treadmill), introduction of low-calorie meals, and intake of laxative (8 pills per day) to accelerate the process of weight loss.

When presenting to the emergency room, the patient was alert and oriented, she had sinus bradycardia (39–40 bpm), her affectivity was flattened and her mood deflected, with marked alexithymia. She had lost 16 kg in 6 months (BMI 14.2) and had been amenorrheic for 9 months. She reported self-injury (nail scratches on the forearms) but denied suicidal ideation (total C-SSRS score 0).

In the initial interview with the child neuropsychiatrist, stubborn refusal of food intake with the declared intent to be hospitalized and desire to lose weight emerged, allegedly not for poor acceptance of her body image, but to try an “extreme experience”, claiming that her life had always been “too simple and free of suffering”. She defined this thought as “perverse” while strongly denying the possibility of death and perceiving herself with “excessive physical strength”. She reported being inspired on Tik Tok, where she saw many teens sharing experiences of deep suffering, often centered on non-suicidal self-injury or eating disorders. She claimed to engage in such conduct with the intent of being hospitalized to demonstrate to herself and others the difficulties of her newly acquired condition.

The patient was admitted to the Department of General Pediatrics of the Bambino Gesù Children’s Hospital, in Rome, where she had weekly interviews with psychologists and neuropsychiatrists specialized in eating disorders. She was fed with enteral nutrition, and then switched over for a period of 4 weeks to feeding entirely by mouth. She was allowed to use the mobile only for education purposes and to go out a couple of hours a day to perform group activities with other patients hospitalized with the same diagnosis. She was initially treated with aripiprazole, as indicated [9,10]. Although a diagnosis of major depression was never made, due to the important mood deflection, it was necessary to treat her with fluoxetine. She demonstrated a strong perseverance in refusing food, even though she was aware of her poor health and the reasons that had led her to that condition. She partially recovered her weight (BMI 15.8) and was no longer bradycardic, thus she was discharged with a specific diet and neuropsychiatric follow-up.

## 3. Discussion

The intent of this article is to show how social media can promote self-injury and eating disorder behaviors. The social media platform referred to in this case is Tik Tok, a platform whose public is primarily made of young adolescents. Unlike other social networks where written content (i.e., Facebook) or images (i.e., Instagram) are favored, Tik Tok provides 15- to 60-second long videos where users usually perform dances to a musical basis [11]. Over time, it has encompassed a wide range of topics, from cooking to performances by professional dancers to political discussions.

The application algorithm records data from the single users and proposes videos that catch the kid’s attention specifically, by creating a personalized “For You” page [12]. This feed will suggest videos from anyone on the platform, not just from the followed accounts [11]. Therefore, if a user accidentally views a video dealing with anorexia on the homepage and, intrigued, searches for other videos alike, the algorithm will keep suggesting such videos, contributing to the development of obsessive behaviors, as in the examined case [12]. These algorithms, with the aim of increasing the diffusion of user-sensitive virtual content, are based on solid models whose development is still ongoing [13]. However, these algorithms are not yet able to discriminate harmless and harmful content, as this, rather than the videos they like, is related to the user’s own life experience, which cannot be embedded in the “formula”.

The theme of anorexia entered Tik Tok through “pro-ana” videos (pro-anorexia), where users supported and encouraged each other to lose weight, exchanging advice and challenging one another [14]. Luckily, most of these videos were blocked by the platform control over content (15.6% of removed videos supported suicide, self-harm, and other dangerous acts) [15], although some still easily circumvent the controls [14,16]. Currently, “anti-pro-ana” (anti-anorexia) videos, officially aimed at raising awareness on anorexia, are much more prevalent [17]. Unlike explicit pro-ana videos, these videos are not subject to banning, with increased content visibility. The same process has also been observed for videos on self-injury [15].

In our case, the patient attended high school with excellent performance and a tendency to perfectionism, she practiced contemporary dance and played cello with a strong commitment. Her family environment was not disrupted, as often occurs in these patients. During the lockdown, with much more free time on hand, she turned to Tik Tok for entertainment and came across videos about eating disorders, non-suicidal self-injury (NSSI) and depression, officially aimed at showing the darker sides of these conditions and scare those who romanticized or promoted them. However, she reported that the video makers are often competitive with one another, as they are tempted to prove they are in the worst condition by showing the numbers, calories, and parameters of “being really sick”. We believe that this kind of content, if shown to fragile teenagers, insecure about their body image, may have a paradoxical effect. In fact, when our patient decided to live such experiences “to grow-up”, she learnt to hide food and vomit, while deceiving others, through these same “good” videos. She then gradually began to lose weight and to self-inflict cuts across her forearms and wrists, until she was admitted to hospital, finally showing herself and others her “true state of suffering”.

Our study has the main limitation of being a case report. As such, it cannot assess a causality relationship between two given events, in our case the exposition to “anti-pro-ana” videos and the development of eating disorders. We therefore hope that further prospective studies may be carried out to better address this putative relationship.

Nevertheless, this is the first article that explores the paradox behind “anti-pro-ana” content on social networks. “Anti-pro-ana communities” have already been mentioned on previous studies, yet always with the intent of demonstrating their positive effect on adolescents [17]. To our knowledge, only one other work approached the same topic, in a different way [18]. In this recently published study, virtual content under the hashtag #Edrecovery (eating disorder recovery) were analyzed, highlighting the link between these and pro-eating disorder videos on Tik Tok [19].

## 4. Conclusions

With this article, we intend to raise awareness on the influence that social media exerts on young adolescents, whose parents are often unaware [16] and whose teachers and pediatricians need to consider more closely. With the restrictions due to the Covid-19 pandemics, the lives of adolescents have radically changed, losing daily habits and sources of leisure and entertainment, and turning to social networks to fight the sense of isolation and boredom [20]. We showed how easy it is to come across messages that are harmful both to the mental and physical health of adolescents on such platforms. In fact, what seems like harmless videos about anorexia or self-injury may instead paradoxically trigger the emulation of such destructive behaviors.

## Data Availability

No new data were created or analyzed in this study. Data sharing is not applicable to this article.

## References

[1] Micali N., Hagberg K.W., Petersen I., Treasure J.L. (2013). The incidence of eating disorders in the UK in 2000–2009; findings from the General Practice Reasearch. BMJ Open..

[2] Löwe B., Zipfel S., Buchholz C., Dupont Y., Reas D.L., Herzog W. (2001). Long-term outcome of anorexia nervosa in a prospective 21-year follow-up study. Psychol. Med..

[3] Pérez S., Marco J.H., Cañabate M. (2018). Non-suicidal self-injury in patients with eating disorders: Prevalence, forms, functions, and body image correlates. Compr. Psychiatry.

[4] Turner P.G., Lefevre C.E. (2017). Instagram use is linked to increased symtomps of orthorexia nervosa. Eat Weight Disord.

[5] Bresnick E. (2019). Intesified Play: Cinematic Study of Tiktok Mobile App. https://www.researchgate.net/publication/335570557_Intensified_Play_Cinematic_study_of_TikTok_mobile_app.

[6] Yokota S. (2020). A Look at TikTok Overtaking the World in 2019. https://en.lab.appa.pe/2020-01/a-look-at-tiktok-overtaking-the-world-in-2019.html.

[7] (2020). 10 TikTok Statistics That You Need to Know in 2021 [Infographic]. https://www.oberlo.com/blog/tiktok-statistics.

[8] Bartz D. (2020). Advocacy Group Says TikTok Violated FTC Consent Decree and Children’s Privacy Rules. https://www.usnews.com/news/technology/articles/2020-05-14/advocacy-group-says-tiktok-violated-ftc-consent-decree-and-childrens-privacy-rules.

[9] Kaye W.H., Wierenga C.E., Bailer U.F., Simmons A.N., Bischoff-Grethe A. (2013). Nothing tastes as good as skinny feels: The neurobiology of anorexia nervosa. Trends Neurosci..

[10] Frank G.K.W., Shott M.E., Hagman J.O., Schiel M.A., Deguzman M., Rossi B. (2017). The partial dopamine D2 receptor agonist aripiprazole is associated with weight gain in adolescent anorexia nervosa. Int. J. Eat. Disord..

[11] Official Website of Tiktok. https://www.tiktok.com/foryou?lang=it.

[12] Tiktok Legal Privacy. https://www.tiktok.com/legal/privacy-policy?lang=it.

[13] Amato F., Moscato V., Picariello A., Sperlí G. Diffusion algorithms in multimedia social networks: A preliminary model. Proceedings of the 2017 IEEE/ACM International Conference on Advances in Social Networks Analysis and Mining.

[14] Marsh S. (2020). TikTok Investigating Videos Promoting Starvation and Anorexia. https://www.theguardian.com/technology/2020/dec/07/tiktok-investigating-videos-promoting-starvation-and-anorexia.

[15] Tiktok Community Guidelines. https://www.tiktok.com/community-guidelines?lang=it#34.

[16] Maloney A. (2020). In 10 Minutes on TikTok I Saw Self-Harm, Girls Offering Sex, Boys Wielding Knives and Potentially Deadly Challenges. https://www.thesun.co.uk/tech/10888684/10-minutes-tiktok-sex-knives-self-harm/.

[17] Oksanen A., Garcia D., Räsänen P. (2016). Proanorexia communities on social media. Pediatrics.

[18] Herrick S.S.C., Hallward L., Duncan L.R. (2020). “This is just how I cope”: An inductive thematic analysis of eating disorder recovery content created and shared on TikTok using #EDrecovery Affiliation. Int. J. Eat. Disord..

[19] Yom-Tov E., Fernandez-Luque L., Weber I., Crain S.P. (2012). Pro-Anorexia and Pro-Recovery Photo Sharing: A Tale of Two Warring Tribes. J. Med. Internet. Res..

[20] Cauberghe V., Van Wesenbeeck I. (2020). How Adolescents use Social Media to cope with feeling of loneliness and Anxiety during Covid 19 Lockdown. Cyberpsychol. Behav. Soc. Netw..

